# GC–MS metabolic profiling of Cabernet Sauvignon and Merlot cultivars during grapevine berry development and network analysis reveals a stage- and cultivar-dependent connectivity of primary metabolites

**DOI:** 10.1007/s11306-015-0927-z

**Published:** 2016-01-23

**Authors:** Alvaro Cuadros-Inostroza, Simón Ruíz-Lara, Enrique González, Aenne Eckardt, Lothar Willmitzer, Hugo Peña-Cortés

**Affiliations:** Max-Planck Institute for Plant Molecular Physiology, Am Mühlenberg 1, 14476 Potsdam-Golm, Germany; MetasysX, Am Mühlenberg 11, 14476 Potsdam-Golm, Germany; Instituto de Ciencias Biológicas, Universidad de Talca, 2 Norte 685, Talca, Chile

**Keywords:** *Vitis vinifera*, Grapevine berry, GC–MS, Grapevine metabolome, Metabolic profiling

## Abstract

Information about the total chemical composition of primary metabolites during grape berry development is scarce, as are comparative studies trying to understand to what extent metabolite modifications differ between cultivars during ripening. Thus, correlating the metabolic profiles with the changes occurring in berry development and ripening processes is essential to progress in their comprehension as well in the development of new approaches to improve fruit attributes. Here, the developmental metabolic profiling analysis across six stages from flowering to fully mature berries of two cultivars, Cabernet Sauvignon and Merlot, is reported at metabolite level. Based on a gas chromatography–mass spectrometry untargeted approach, 115 metabolites were identified and relative quantified in both cultivars. Sugars and amino acids levels show an opposite behaviour in both cultivars undergoing a highly coordinated shift of metabolite associated to primary metabolism during the stages involved in growth, development and ripening of berries. The changes are characteristic for each stage, the most pronounced ones occuring at fruit setting and pre-Veraison. They are associated to a reduction of the levels of metabolites present in the earlier corresponding stage, revealing a required catabolic activity of primary metabolites for grape berry developmental process. Network analysis revealed that the network connectivity of primary metabolites is stage- and cultivar-dependent, suggesting differences in metabolism regulation between both cultivars as the maturity process progresses. Furthermore, network analysis may represent an appropriate method to display the association between primary metabolites during berry developmental processes among different grapevine cultivars and for identifying potential biologically relevant metabolites.

**Electronic supplementary material:**

The online version of this article (doi:10.1007/s11306-015-0927-z) contains supplementary material, which is available to authorized users.

## Introduction

Grape is a non-climacteric fruit and one of the most important crop in the world. Approximately 7.400.000 ha are dedicated to its cultivation worldwide, with an estimated production of 67Mt (FAO 2008, http://faostat.fao.org/site/567/default.aspx). Around 71 % of this production is used for wine, 27 % as fresh fruit, and 2 % as dried fruit (raisins). Grape growth, development and ripening has been widely studied in the literature due to the particular characteristics of the processes in this plant species and the interest to understand the physiological and biochemical events that determine grape and wine quality (Coombe and McCarthy 2000; Conde et al. 2007; Agudelo-Romero et al. 2013a; Kuhn et al. 2014).

In the last few years, multiple studies mainly based on transcriptomic analysis and some other works on proteomics or integration of omics data have provided information at molecular levels of genes and proteins involved in the multiple physiological and biochemical events which may determine growth, development and ripening of grapevine berries (Waters et al. 2006; Pilati et al. 2007; Deluc et al. 2007; Deytieux et al. 2007; Grimplet et al. 2009; Zenoni et al. 2010; Zamboni et al. 2010; Fasoli et al. 2012; Torniellei et al. 2012; Rienth et al. 2014).

Wine composition and quality is determined by a number of parameters reflecting variability due to the source of the biological material used (most prominent the grape variety/maturity and the yeast strain) as well as variability inherent in the various processing steps performed during the wine making process and storage. Thus based on the main requirements by controlling both the processing and the biological material, the wine-makers should be able to have access to management of wine quality in a highly standardized way. Whereas the post-harvest processing steps can be fairly well controlled, the control of the biological starting material, notably the berries with respect to parameters such as berry size, sugar content, optimal harvest time, content of anthocyanins, phenolic, and volatile compounds which have been found to be essential for wine quality, is much more difficult to achieve. In addition to being determined by the grapevine variety these parameters are strongly dependent on the exact environmental conditions (soil, temperature, humidity, UV radiation, etc.) and agronomics practices (Kliewer and Dokoozlian 2005; Lund and Bohlmann 2006; Holt et al. 2008). A comprehensive understanding of the diverse physiological and biochemical processes involved in the biological stages of grape fruit formation, development and ripening should help achieving better control of this process ultimately leading to an improvement of grape berry quality and, in consequence, wine quality.

Grape development from flowering to ripening is usually divided in three phases based on morphological and biochemical changes (Coombe and McCarthy 2000): (i) early fruit development, characterized by exponential growth and accumulation of solutes such as tartaric and malic acid; (ii) veraison, a stage mainly characterized by berry color change and growth reduction; and (iii) ripening, by resuming growth and accumulation of sugars, anthocyanins and flavor compounds. There are several studies at the transcript level describing the underlying changes on the gene expression level based on either EST sequencing or array hybridization approaches either considering different developmental stages or some specific tissues of grape berries (Peng et al. 2007; Pilati et al. 2007; Zenoni et al. 2010, Fortes et al. 2011; Guillaumie et al. 2011; Lijavetzki et al. 2012, Diaz-Riquelme et al. 2012; Sweetman et al. 2012; Agudelo-Romero et al. 2013a). With respect to proteomics, several studies have characterized protein levels in different grape tissues, such as flesh, fruit and skin, as well as wines, under different stress conditions and different stages of ripening (Grimplet et al. 2009; Palma et al. 2011; Sharathchandra et al. 2011; Martínez-Esteso et al. 2013). Large efforts have been dedicated to characterize the chemical components of grapevine tissues. However, those studies have been performed in a classic targeted approach mostly focused on the specific class of compounds, such as organic acids malic and tartaric acid, phenolics, volatile compounds or plyamines (Ribéreau-Gayon et al. 2000; Oliveira et al. 2004; Adams 2006; Kennedy et al. 2006; Teixeira et al. 2013; Agudelo-Romero et al. 2013b).

More comprehensive studies on the metabolite level have been reported in the last few years by using different platforms available for the high throughput analysis of plant metabolites and metabolomes, varying in their selectivity and sensitivity. However, most of such studies are limited to the metabolome analysis of grapevine berries of a determined cultivar and/or some few berry developmental stages (Deluc et al. 2007; Grimplet et al. 2009; Zamboni et al. 2010; Fortes et al. 2011), to a certain plant tissue (Lawo et al. 2011) or to a particular kind of stress condition (Hong et al. 2012). More comprehensive and comparative studies focused on the characterization of grapeberry development have been recently reported considering diverses grapevine cultivars, several developmental berry stages/tissues, or combining different “omics” platforms in the analysis (Torniellei et al. 2012). For instance, Ali et al. (2011) described biochemical changes during four grape berry development stages of five grape cultivars from Portugal by NMR spectroscopy, while Dai et al. (2013) perfomed a metabolomic description of 10 different developmental phases of a specific clone of Cabernet Sauvignon (CS) by using LC–MS from either field-grown vines or fruiting cuttings grown in the green-house. Metabolic profiles during late berry development in the Italian grapevine cultivar Corvina have been also reported (Toffali et al. 2011) as well metabolic phenotyping of berries in different grape cultivars (Son et al. 2014) or of grapes of controlled appellation regions (Teixeira et al. 2014). Some other studies advise omics data integration of transcriptomics, proteomics and metabolomics during grape development for the cultivar Corvina (Zamboni et al. 2010), the cultivar Trincadeira (Fortes et al. 2011) and Aragones and Touriga Nacional (Agudelo-Romero et al. 2013a).

Grape berry development involves changes in size and and composition. From being small, firm, and acidic with little sugar and desirable flavours or aroma the berries turn into larger, softened, sweet, highly flavoured, less acidic, and highly coloured fruit. The development of these characteristics determines the quality and attributes of the final fruit. Primary metabolites are key components and are playing a crucial role during the different stages of berry development and the generation of flavor and aroma properties. For instance, during the first growth period chlorophyll is the main pigment present in fruit which are also rich in organic acids such as tartaric and malic acids. Thereafter, a huge increase of sugars like glucose and fructose is observed as well as an augmentation of phenolic and aromatic compounds, whereas malate content declines (Davies and Robinson 1996). On the other hand, the balance acid-sugar partially determines the development of flavour in table grapes (Boss and Davies, 2001) while aromas arise from volatile compounds such as terpenes, norisoprenoids, and thiols stored as sugar or amino acid conjugates (Lund and Bohlmann 2006). Thus, the correct ripening of the grape berry is fundamental for both the commercial value of the fruit (table grape) and the quality of wine.

In consequence, changes in chemical substances (metabolites) levels are playing a role in both growth and maturity of the grape berry but it is still unclear to what extent primary metabolite modifications differ between cultivars during ripening as well it is still unknown how such metabolites are connected or interacting during development and ripening stages in different grape vine cultivars. In order to gain a broader and deeper insight on metabolic composition of grapevine berry development and to evaluate the extent to which metabolite levels vary between wine grape cultivars and how they are interacting with each other during ripening of the berries, we performed a comparative non-targeted metabolic analysis by a high-throughput metabolic profiling platform based on GC–MS technology of six developmental stages of grape berries from field-grown vines, starting with flowers and finishing with mature berries samples of the cultivars CS and Merlot (ME). Multivariate tools were utilized to integrate and explore the measured data. In addition to comparing the resulting data directly via relative concentrations of individual compounds, a more integrated view based on metabolite–metabolite correlations by using a network approach was used (Fukushima et al. 2011; Sakurai et al. 2011; Sweetlove and Fernie 2005; Toubiana et al. 2013).

## Materials and methods

### Plant material

Grapevine (*V. vinifera* cv CS and ME) berries at different stages of development were collected from vines located in Colchagua Valley, Chile, during two consecutive field seasons (2008–2009 and 2009–2010). Corresponding to their developmental stage, collected berries were separated from their clusters and around 10–50 berries were pooled together to produce a biological sample. We did not separate berry tissues, such as flesh, skin and seed. We grouped the collected grapevine berry samples into six developmental stages according to Coombe (1995) (see Supplementary Fig. 1), where each stage composed of around ten biological samples for each cultivar, five samples per year. Phenological stages analyzed and their corresponding numbers according to the ‘Modified Eichorn-Lorenz classification system’ (Coombe 1995) and samples from each cultivar were collected during the indicated days post-anthesis (DPA). Flowering (EL-23, 0 DPA), Fruit setting, (EL-29; Cabernet Sauvignon: 16 DPA; Merlot: 19 DPA), Pre-veraison, (EL-33; Cabernet Sauvignon: 43 DPA; Merlot 39 DPA), Veraison, (EL-35; Cabernet Sauvignon: 52 DPA; Merlot: 44 DPA), Post-veraison, (EL.36; Cabernet Sauvignon: 60 DPA; Merlot: 50 DPA) and Ripening (EL-38; Cabernet Sauvignon: 115 DPA; Merlot: 103 DPA). After collection, samples were immediately frozen in liquid N_2_ and stored at −80 °C until processing. For the purpose of metabolite analysis as well as metabolic network reconstruction, we decided to consider these 6 stages as reference points and compare them to each other between the two cultivars studied.

### Metabolite extraction and analysis by GC–MS

Metabolic extraction and derivatization of metabolites from whole grape berries for GC–MS analysis were performed by using a modified method of the one previously outlined by Lisec et al. (2006). 15 mg of fresh tissue were mixed with 1 mL of extraction buffer (pre-cooled at −20 °C), containing H_2_O, MeOH, CHCl_3_ (1:2.5:1), and vortexed for 10 s. Deuterated cholesterol and 13C sorbitol were spiked in the extraction buffer as internal standards in order to identify potential chromatographic errors. Mixtures were subsequently placed in a shaker for 5 min at 4 °C. The homogenized material was centrifuged at 14000 rpm for 2 min and the supernatant transferred to new tubes. 400 µL of pure water were added to the supernatant, vortexed, and centrifuged at 14,000 rpm for 2 min. The polar (upper) phase was transferred and divided in two aliquots, one for metabolite measurements, and the other was kept as back-up and stored at −20 °C. A portion of the first aliquot was diluted in a ratio of 1:20 for a second injection to allow measurement of highly abundant sugars. Both aliquots were dried out in a vacuum concentrator without heating. The first aliquot was dried in a vacuum concentrator without heating. The derivatization protocol was performed as described by Lisec et al. (2006). Sample measurement order was randomized in order to avoid experimental drifts. GC–MS data were obtained using an Agilent 7683 series autosampler (Agilent Technologies GmbH, Waldbronn, Germany), coupled to an Agilent 6890 gas chromatograph—Leco7 Pegasus 2 time-of-flight mass spectrometer (LECO, St. Joseph, MI, USA). Identical chromatogram acquisition parameters, as those previously described, were used (Weckwerth et al. 2004).

### Metabolite data pre-processing and statistical analysis

Raw GC–MS chromatograms were imported to Leco ChromaTOF software (version 3.25), baseline corrected, and exported to machine independent network Common Data Form (netCDF) files. Peak-picking, retention time alignment and metabolite library search were performed by the *TargetSearch* package from bioconductor (Cuadros-Inostroza et al. 2009) with the R environment (http://www.r-project.org). Metabolites were manually annotated by using Leco ChromaTOF software against an in-house reference library: The Golm Metabolome Database (GMD@CSB.DB, Hummel et al. 2007). Metabolite data were normalized by dividing each raw value by the median of all measurements of the experiment for one metabolite. After that, the data were log2 transformed before performing statistical analysis. All data manipulation and statistical tests were performed by using freely-available packages together with custom R scripts. Briefly, hierarchical clustering analysis (HCA) was performed using Euclidian distance and complete linkage on the metabolite levels. Before performing HCA, we computed the median level of each metabolite across replicates for each developmental stage and cultivar. Principal component analysis (PCA) was carried out using the package pcaMethods (Stacklies et al. 2007). The data were centered and unit-variance scaled before the PCA computations. Partial least squares (PLS) discriminant analysis was performed using the package mixOmics (Lé Cao et al. 2009). The data was centered at unit-variance scale, two components were included in the models and they were validated by leave-one-out cross-validation. To compare significant changes across stages, we performed ANOVA first and, in case the result ensued as significant, we applied Tukey Honest significant differences to determine what stages were significantly different from the others. To avoid multiple-testing problems, we corrected the resulting p-values by the false discovery rate method (Benjamini and Hochberg 1995). In all tests, we considered adjusted p-values lower than 0.05 as significant.

A simplificated metabolic pathway of primary metabolism was created to allow simultaneous visualization of all grape developmental stage data, which was based on maps taken from MapMan software (Usadel et al. 2009). A custom python script was used to import the metabolite data matrix into the pathway.

### Network analysis

A metabolite network was constructed for each possible cultivar-developmental stage combination, twelve networks in total (two cultivars, six stages). For each network, 10 samples and all measured metabolites were considered. The procedure was the same for each network. The data workflow is depicted in Supplementary Fig. 2. Firstly, metabolite–metabolite correlation analysis was performed by using Spearman’s rank correlation coefficient. To estimate the correlation significance, we used the following bootstrap method in which the metabolite matrices were first randomly shuffled without keeping column or row orders and then the correlations were computed. This procedure was repeated 1000 times and a *p* value was calculated based on the frequency of the observed *r*-values. The resulting *p*-values where further corrected by applying the Benjamini–Hochberg method (Benjamini and Hochberg 1995). We used |*r|* > 0.75 as a correlation threshold for all networks, since the observed *p*-values were lower than 0.001.

Correlation matrices were first transformed to adjacency matrices by applying the previously established correlation threshold. Network analysis was performed by using custom scripts within the R environment (http://www.r-project.org) and *Cytoscape* (http://www.cytoscape.org/). The R-package *igraph* (Csardi and Nepusz 2006) was used mainly for network manipulation and parameter calculation, whilst *Cytoscape* was used for visualization. All network parameters were obtained by using the respective *igraph* function, except the *network cluster coefficient*, which was calculated as the average of the cluster coefficients (*Cn*) of its nodes (Dong and Horvath 2007). Network topologies were analyzed by applying a goodness-of-fit test for exponential and power-law distributions. Non-linear least squares models were fitted to the cumulative degree distribution of every network. Akaike’s information criterion was used to obtain the likelihood of the fitted models.

To estimate network overlap significance, we used two methods: Fisher’s exact test and network randomization. In the latter case, two networks to be compared were randomly rewired but conserving their original degree of distribution. This was repeated 1000 times and the observed frequency of edge overlap was recorded. An estimated *p*-value was calculated based on the probability of obtaining the original overlap by chance. Similarly, the significance of average path difference between two networks was estimated by using the empirical average-path difference distribution of the randomization procedure.

## Results

### Structure of the experiment and data extraction

To gain information about primary metabolites during the whole development process of grapevine berries, we measured metabolite levels of two grape berry cultivars (CS and ME) by using a metabolomics platform based on gas chromatography–mass spectrometry (GC–MS) of polar extracts and the R-package *TargetSearch* (see Sect. 2) to identify the metabolites. The Golm Metabolome Database (GMD@CSB.DB, Hummel et al. 2007), an in-house metabolite database comprising around 950 metabolites (Kopka et al. 2005), was utilized as a reference library to search for metabolites. In total, 115 metabolites were obtained by this algorithm, which were subsequently curated by manual inspection (Supplementary Table 1). Metabolites were classified by chemical classes as given by the GMD (Table 1). Considering the classified metabolites, the most represented metabolite classes were miscellaneous acids (21), sugars (19), and amino acids (19). Metabolites that were partially annotated were classified as unknown (20), and those that do not belong to any of the other metabolite classes, according to the GMD, were denominated as unclassified metabolites. The miscellaneous acids class comprises different acid types, among those, dicarboxylic acids, hydroxy acids, phenylpropanoic acids, and hexonic acids were the most represented.

**Table 1:** Number of metabolites within each class

Metabolite class	Total identified metabolites
Amino acid	19
Fatty acid	4
Acid	22
Flavonoid	3
Miscellaneous	11
Sugar	19
Unclassified	17
Unknown	20
Total	115

The acid class comprises different acid sub-classes that had few members each, among those, aromatic acids, hydroxy acids, dicarboxylic acids, phenylpropanoic acids. Similarly, the miscellaneous class includes other compound classes which did not fit in any other main classes and had few representatives, such as amides, polyols, pyrimidine, and terpenoides. Unclassified metabolites are known metabolites that are not annotated in a chemical class according to the GMD database

### PCA analysis allow a separation of early and late developmental stages in both cultivars

**Fig. 1:**
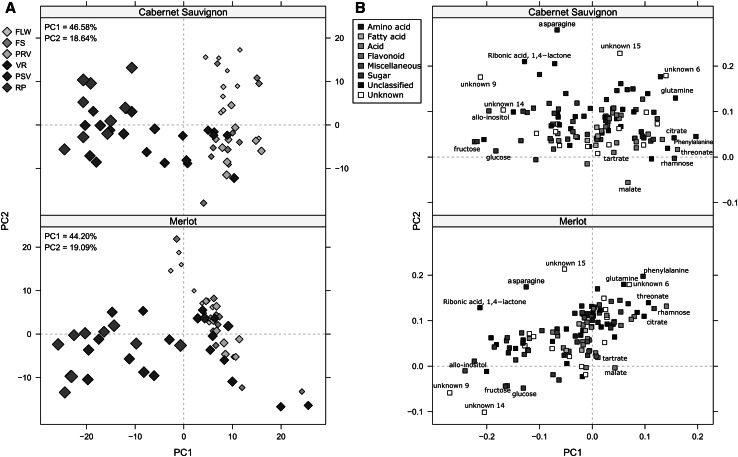
PCA of primary metabolites of grape whole berry samples. **a** PCA scores of the 115 primary metabolite data measured by GC–MS. Grape cultivars are represented by a different *panel* as shown. Developmental stages are indicated by different *diamon*d sizes and *colors*: *FLW* flowering, *FS* fruit setting, *PRV* pre-veraison, *VR* veraison, *PSV* post-veraison, *RP* ripening. The explained variances of principal components are shown in the upper-left corner. **b** PCA loadings of the primary metabolite data. Metabolite classes are color coded as shown in the *upper-right panel*. Metabolites that have a major influence in the separation are named (Color figure online)

**Fig. 2:**
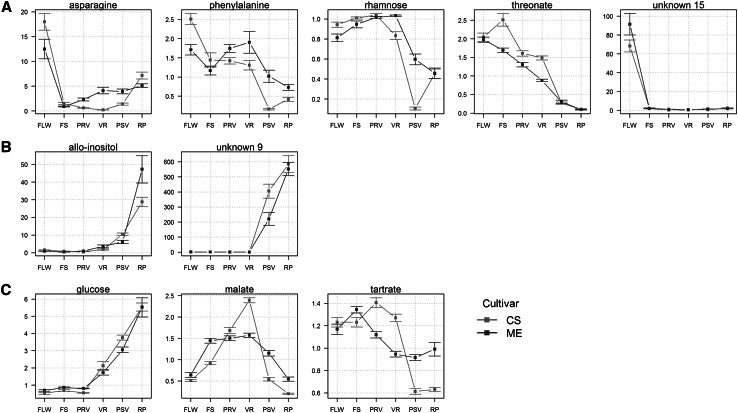
Metabolite levels of main discriminating factors of the PCA (Fig. 2) in Cabernet Sauvignon (CS, *red line*) and Merlot (ME, *blue line*). Time points correspond to the six developmental stages: *FLW* flowering, *FS* fruit setting, *PRV* pre-veraison, *VR* veraison, *PSV* post-veraison, *RP* ripening. Data are normalized to the median intensity across all the samples. Values represent the mean ± standard deviation. Metabolites are put into groups according to their profiles: **a** accumulation in early stages, **b** accumulation in late stages, and **c** known metabolites that change during growth but had lower discriminating power (Color figure online)

To have an overview of the data, we performed PCA on every cultivar data set (Fig. 1). Considering only the first two principal components, the explained variance was similar in both cultivars, accounting for 65.22 % in CS, and 63.29 % in ME (Fig. 1a). A clear separation between the earliest and latest developmental stages is observed for both cultivars, however the early stages of development are not well differentiated between both cultivars. The first two stages (flowering and fruit setting) are better separated in CS (Fig. 1a, upper panel) when compared to ME, where only flowers show some separation, whilst fruit-setting, pre-veraison, and veraison are overlapping. Since the samples were harvested during two growth seasons, we asked whether there is a year effect reflected on the PCA. However, no separation was observed (data not shown), therefore we decided to disregard the year factor and have combined the results of both years. The metabolites that drive the stage separation can be observed in the PCA loading plot (Fig. 1b). Although these metabolites are the same in both cultivars, they display different behavior in each of them. For instance, in CS (upper panel), the main contributing factor of principal component two is asparagine, followed by unknown metabolite 15. In ME (lower panel Fig. 2b), the role is inverted: unknown metabolite 15 is the most important factor followed by asparagine. Among the factors that drive the separation of the first principal component, which differentiate between early and late developmental stages, unknown metabolite 9, allo-inositol, fructose, glutamine, phenylalanine, threonate, and rhamnose can be identified. These metabolites exhibit a similar role in stage separation in both cultivars and follow two opposite concentration patterns. On one side, asparagine, phenylalanine, rhamnose, threonate and unknown 15 accumulate during the early stages and their levels are much lower in berries during ripening (Fig. 2a), while others like allo-inositol and metabolite unknown 9 accumulate at later stages (Fig. 2b). Well known metabolites present in grapevine berries that change their levels during growth such as glucose, tartrate and malate had a rather minor discriminating power (Fig. 2c).

### Discrimination of cultivars by developmental stages

PCA analysis of the determined metabolites revealed the presence of discriminating factors which allow the separation of the different developmental stages of a given cultivar (Fig. 1). Performing PCA for discriminating the same developmental stage betweeen both cultivars did not allow to observe any separation of the samples by developmental stages in any of the respective score plots (data not shown), although in some stages, a separation was indeed observed in higher principal components.

To verify whether our metabolic analysis provided enough information allowing the discrimination of each developmental stage when both cultivars are compared we decided to use a supervised method as PLS. It turned out that this method does allow the discrimination of both cultivars in every developmental stage (see Supplementary Fig. 3). In order to find the discriminant factors, we looked for significant differences (*p* < 0.05. twofold-change) of the metabolite concentrations in each dataset (see Supplementary Table 2). These results correlate with main loadings of the PLS regression (see Supplementary Table 3). In post-veraison the most significant differences were observed (12 significant changes, see Supplementary Table 4). Many amino acids such as gaba, serine, methionine, ornithine have higher mean concentrations in ME compared to CS. Fruit setting was the stage where fewer disparities between the cultivars were observed, only citrate and unknown metabolite 18 were up-regulated in CS, while glycolate was up-regulated in ME. Almost all metabolites displayed differences in one or two stages, while only two metabolites, galactinol and glycolate, showed differences in three stages. This indicates that while there are metabolites which discriminate both cultivars, these are only stage dependant, since no metabolite was consistently different across the whole growth period.

### Sugars and amino acids levels show opposite behaviour during berry maturation

In order to illustrate the PCA results, we carried out HCA on the data in order to identify metabolite patterns across the developmental stages. To facilitate the pattern visualization, we kept the order of the samples fixed, from early to late stages, in both cultivars. The HCA reveals that, overall, the two cultivars have similar metabolite patterns with particular small differences in the distribution of some compounds (see Supplementary Fig. 4). We identified the following patterns that are summarized in Supplementary Fig. 5. The first cluster is composed of metabolites that increase their abundance along the growth curve during post-veraison and ripening, most of them being sugars and some amino acids such as proline, β-alanine, and asparagine (see Supplementary Fig. 5A). The second cluster comprises metabolites that decrease during grape development, which contains mostly amino acids and polyamines related compounds (see Supplementary Fig. 5B). An important observation is that not all type of sugars show an increase during grape development, as can be observed with ribose, arabinose, rhamnose, and fucose (see Supplementary Fig. 5B). The HCA also shows that the metabolites that contribute to the distinction of the growth stages, as discussed previously in Fig. 1b, exhibit different increasing (unknown metabolite 9, allo-inositol, and fructose; see Supplementary Fig. 5C) and decreasing (glutamine, phenylalanine, threonate, and rhamnose; see Supplementary Fig. 5B) patterns, as also deducible from the PCA where they lie in opposite directions along principal component 1 (Fig. 1b).

### Fruit-setting and veraison are accompanied by the largest number of changes in metabolite concentration

**Fig. 3:**
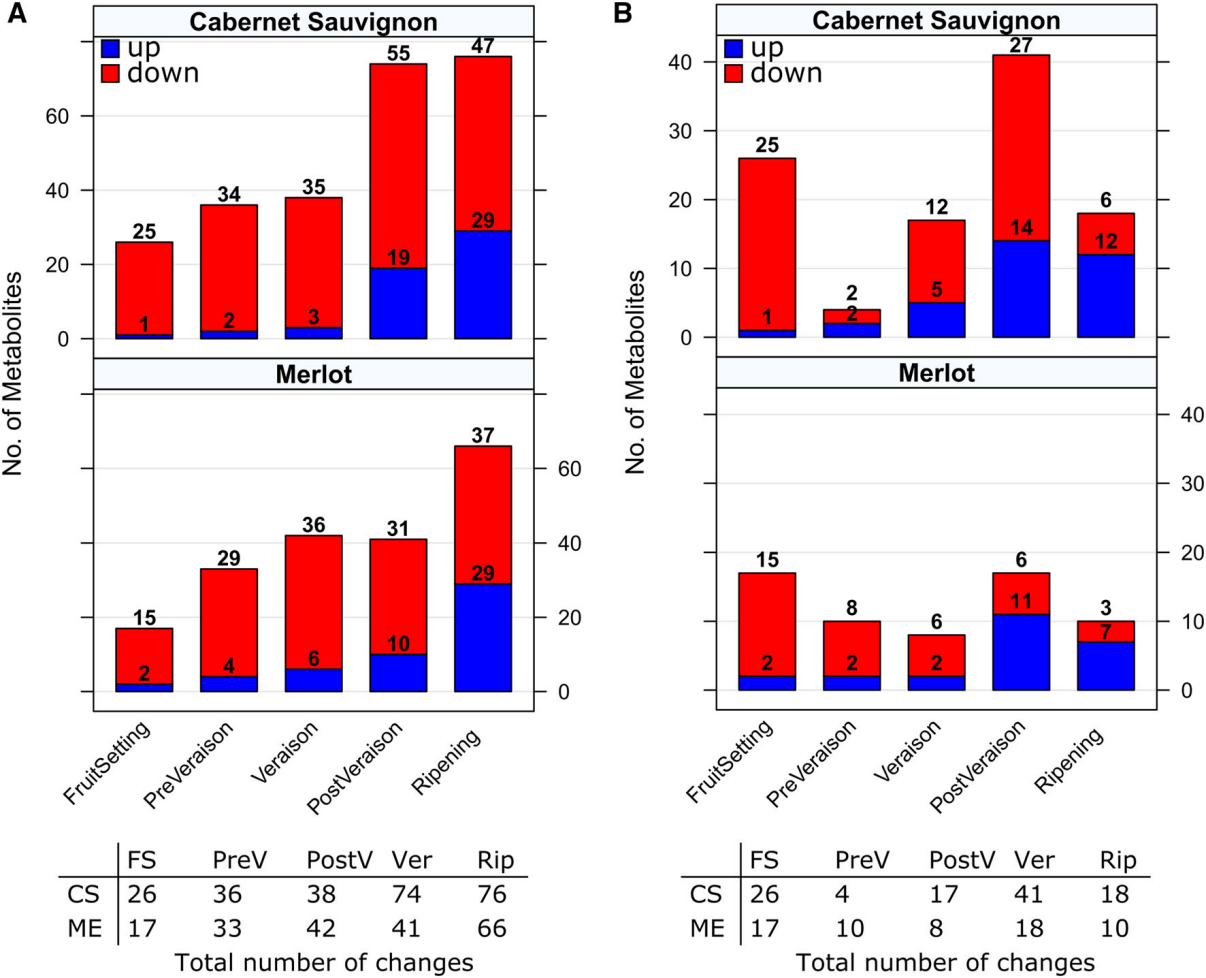
Number of significantly changing metabolites across developmental stages. **a** Each panel shows the number of significantly changing metabolites (*p* < 0.05, ANOVA) with respect to the first stage (flowering) across developmental stages for Cabernet Sauvignon (*upper panel*) and Merlot (*lower panel*). **b** Number of significant changes (*p* < 0.05, ANOVA) with respect to the previous stage for each cultivar, that is, fruit setting vs. flowers, pre-veraison vs. fruit settings, and so on. Significantly increasing and decreasing metabolites are represented by *blue* and* red bars*, respectively. In both figures, the tables shown below summarize the total number of changes (*up plus down*) for each cultivar (Color figure online)

Berry ripening is accompanied by massive biochemical and physiological developmental changes which likely are also accompanied by major changes on the metabolite level. We thus examined the magnitude of metabolites changes expressed in accumulation (up-regulated) or reduction (down-regulated) of them during berry development comparing: (i) the metabolites variation of each stage to the initial developmental stage (flowering, FLW) (Fig. 3a) and (ii) the metabolite changes of a given stage to the preceding ones (Fig. 3b). Contrasting the number of metabolites changing in each stage to the initial developmental stage (flowering), we observed that the number of metabolites decreasing their concentration (down-regulated) is higher than those increasing their concentration (Fig. 3a). This behavior is exhibited in both cultivars. The number of down-regulated metabolites increases when the latest developmental stages (post-veraison and ripening) are compared to the stage of flowering (55 and 47 in CS, and 31 and 37 in ME). Similarly, an increase of the number of metabolites which accumulate (up-regulated) during the development of grapevine berries was detected, starting with 1 and 2 metabolites in CS and ME in the transition of flowers to fruit setting and reaching 29 metabolites in both CS and ME, respectively in ripening stages (Fig. 3a). Evaluation of metabolites variation by comparing certain stage to the directly previous ones allowed to establish which of the stages shows the largest number of changes in metabolism as compared to the previous growing stage during grapevine berry development. The results in Fig. 3b show some differences between both cultivars regarding to the down- and up-regulated metabolite changes. While the largest number of down regulated changes occurs in the transition to fruit setting, veraison and post-veraison in CS, in ME they materialize in the transition to fruit setting, pre-veraison and veraison. Up-regulated metabolite alteration displays a similar behaviour in both cultivars, increasing metabolites content in post-veraison and ripening.

Thus, the results comparing either the metabolites changes of each developmental stage to metabolic profile of flowers or to the directly previous ones suggest that the largest biochemical changes going on during grapevine berry development involved the reduction of a series of compounds already present in the initial stages (flowering), which essentially occurs in the switch to fruit setting, and an accumulation of other substances which take place in post-veraison and ripening in both cultivars.

To identify common regulated metabolites between both cultivars for every developmental stage, pair wise overlaps of increasing and decreasing metabolites were computed as well as the overlap between the two cultivars. Results displayed as Venn diagrams (see Supplementary Fig. 6A and B) demonstrated the presence of common molecules which are increasing or decreasing in a certain developmental stages in both cultivars as well other which are present in only one of the cultivar in a giving growth stage (cultivar-specific substances).

Among the metabolites displaying a significantly increase in fruit setting, pre-veraison and veraison and similar behaviour in both cultivars was malate. Glucose, fructose, glucaric acid-1,4-lactone, beta-d-fructofuranosyl, and three unknown metabolites increased significantly in post-veraison as well as ripening in both cultivars. Many amino-acids decreased during growth, such as phenyl-alanine, serine, and valine, as well as polyamines (spermine and spermidine) in almost all stages.

In post-veraison, metabolites that increase their abundance are mostly sugars: fructose, glucose, and glucopyranoside, 1-*O*-methyl. On the other hand, the majority of down-regulated metabolites are acidic substances and other kinds of acids (mainly hydroxy and dicarboxylic acids). However, only three metabolites exhibited significant down-regulation: glycerate, succinate and threonate in both CS and ME (see Supplementary Tables 2 and 5).

### Overview of metabolic changes by simplified pathway representation

**Fig. 4:**
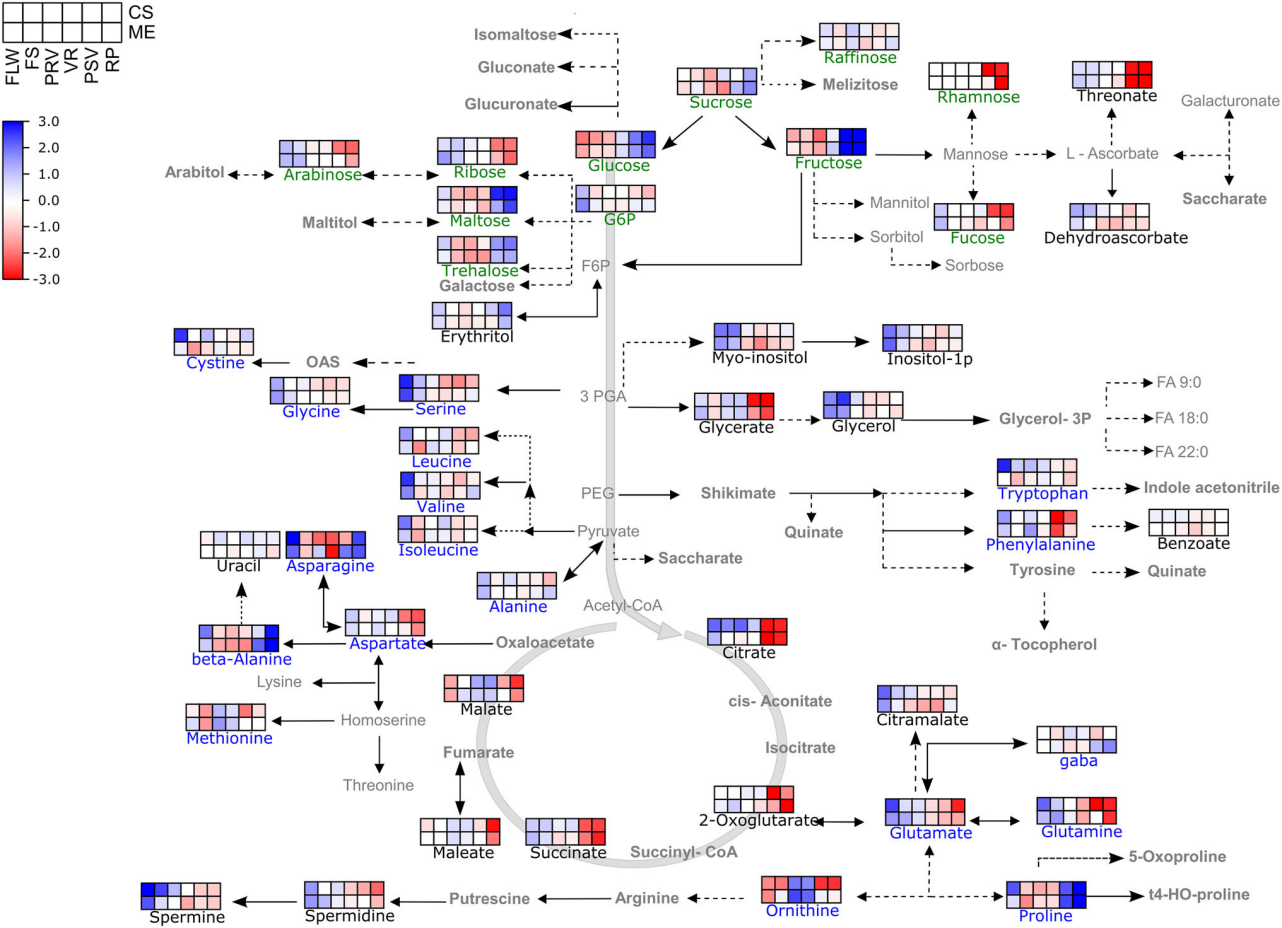
Primary metabolism pathways of measured metabolites Cabernet Sauvignon (CS) and Merlot (ME). Represented pathways are simplified versions of the tricarboxylic acid (TCA) cycle, glycolisis, amino acid synthesis, and sucrose synthesis. Within each *box*, *rows* represent cultivars (*upper row*: CS; *lower row*: ME) and each column is a grape developmental stage (from *left* to *right*: flowering, FLW; fruit setting, FS; pre-veraison, PRV; veraison, VR; post-veraison, PSV and ripening, RP) as shown in the *upper left corner*. Average metabolite intensity is color coded according the scale in the *upper left* *corner*. Amino acids, sugars, and non-measured metabolites are displayed in *blue*, *green*, and *gray* colored font, respectively (Color figure online)

In order to obtain a more detailed overview about the abundance of similarities and/or differences of the identified substances in each developmental stage of both cultivars, we compared the behaviour of some of the metabolites in a simplified versions of primary metabolism pathways by using Map Man tools (Usadel et al. 2009). Figure 4 displays the observed changes by pathway mapping. In sucrose metabolism, sucrose, fructose, and glucose exhibited a similar increasing trend observed in the two cultivars during the different developmental stages. In the TCA cycle, both cultivars basically exhibit similar metabolite profiles, however some differences could be observed: citrate and succinate significantly decreased after veraison in both cultivars; but citrate decreased in ME already in fruit setting, which was not observed in CS. Malate showed an conserved tendence in both cultivars, increasing in fruit setting and decreasing to normal levels in post-veraison. However, a more significant decrease is observed in CS in ripening stage.

With respect to amino acids content, most amino acids such as serine, glycine, leucine, valine, isoleucine, and alanine decreased their level in comparison to the amount detected in the first stage. Only few amino acids display an increase during grape development. Among them, ornithine increased in pre-veraison and veraison in both cultivars while β-alanine, asparagine and proline rise in post-veraison and ripening phases. A unique observation in ME is that GABA increased during berry ripening, which was not observed in CS (also seen in Supplementary Table 4).

The polyamines spermidine and spermine exhibited a similar decreasing trend in both cultivars. In particular, spermine significantly decreased after flowers in both cultivars, whereas spermidine started to decrease in veraison in both CS and ME.

### Network analysis shows that fruit setting stage displays the highest network density

In addition to comparing the resulting data directly via significant relative concentrations changes of individual compounds, we decided to use a more integrated approach based on metabolite–metabolite correlation networks (CNs). A common characteristic frequently found in biological networks is that their degree distribution follows a power law (Watts and Strogatz 1998; Albert and Barabasi 2000; Arita 2005). These types of networks, also called scale-free, are characterized by possessing highly connected nodes (hubs) and that most nodes can be reach from other nodes by a small number of steps, and subsequently they normally display low average shortest path length. To test whether the reconstructed networks were scale-free, we fitted exponential and power law distributions to the corresponding degree distribution (see materials and methods), and based on the resulting p-values (*p* < 0.0005), both distributions were very likely. However, a further analysis by Akaike’s information criterion score suggested that power-law distributions provided a better fit. This result may suggest a biological organization of the reconstructed grapevine metabolic networks, in which few metabolites, hubs, are essential for the network connectivity (Jeong et al. 2001; Giot et al. 2003). The data analysis considering two parameters from the basic network properties calculated (Table 2) such as the number of edges and network density, revealed that within each cultivar, networks associated to fruit setting exhibit by far the highest density and biggest number of edges, and in consequence, they also possess the lowest number of isolated nodes and the smallest diameters. Higher network density can be interpreted as a more constrained metabolism which would suggest a higher metabolic control during this major developmental shift (Supplementary Fig. 7). Early developmental stages networks, namely flowers and fruit setting, have higher density and cluster coefficient, in comparison with the later stages (post-veraison and ripening) in the selected cultivars. The middle stages (pre-veraison and veraison) lie somehow in between regarding density with the ME veraison network being the only exception (Supplementary Fig. 7). All networks exhibit a small average path length, which is in agreement with said property of scale-free networks (Table 2).

**Table 2:** Network properties of metabolite networks per developmental stage. The number of edges and network density reveal that network associated to fruit setting exhibit by far the highest density and number of edges. High network density can be interpreted as a more constrained metabolism which would suggest a higher metabolic control during this developmental shift. See Materials and Methods for details

Cultivars	Developmental stages	Connected nodes	Isolated nodes	Edges	Connected components	Diameter	Average path length	Network density	Cluster coefficient
Cabernet	Flowers	70	45	154	9	9	3.341	0.023	0.252
Sauvignon	FruitSetting	94	21	625	3	7	2.463	0.095	0.424
	Pre-Veraison	66	49	86	9	9	3.889	0.013	0.158
	Veraison	55	60	66	11	6	2.637	0.010	0.122
	Post-Veraison	67	48	72	15	9	2.660	0.011	0.142
	Ripening	62	53	67	17	5	1.725	0.010	0.133
Merlot	Flowers	79	36	183	7	8	3.489	0.028	0.270
	FruitSetting	89	26	455	3	8	2.945	0.069	0.366
	Pre-Veraison	59	56	61	15	6	2.802	0.009	0.149
	Veraison	76	39	276	5	9	3.417	0.042	0.285
	Post-Veraison	62	53	86	13	7	3.129	0.013	0.145
	Ripening	59	56	52	15	6	2.294	0.008	0.093

### A significant proportion of edges is conserved between different developmental stages

Despite the fact that the stage specific networks differ in general network properties such as network densities, there is a significant overlap between the metabolite networks. In CS, the overlap ranges from 3 to 35 %, depending on the stage taken as reference (see Supplementary Table 6A). Although these percentages are rather low, they were highly significant as demonstrated by Fisher’s exact test (*p* < 0.001). Few exceptions of less significant overlaps are flowers versus ripening, and pre- versus post-veraison (both *p* < 0.1). The only non significant overlap was observed between flowers and post-veraison. In ME, all network overlaps are significant, with the exception (and different from CS) of ripening versus pre-veraison and veraison (see Supplementary Table 6B).

### Most abundant sugars such as glucose, fructose and sucrose are of lower importance as based on centrality measures

To identify the most important metabolites as a function of the variety and developmental stage, we performed a common approach to answer this question by determining the so called centrality measures (Dong and Horvath 2007). A simple centrality measure is the degree of the node, which counts the number of its connections. A more sophisticated measure is the betweenness centrality. Betweenness centrality of a node *n* is defined as the proportion of shortest paths between two other nodes that pass through *n* (Brandes 2001). By using these centrality measures, we identified metabolites that exhibited high degree and betweenness in every network. The metabolites displaying the highest betweenness centrality are displayed in Supplementary Table 7A (a full list for all metabolites is shown in Supplementary Table 8). As a general result we observed that significant metabolite abundance changes do not correlate with high betweenness or node degree again emphasising once more that network analysis and single compound centered analysis identify different properties.

**Fig. 5:**
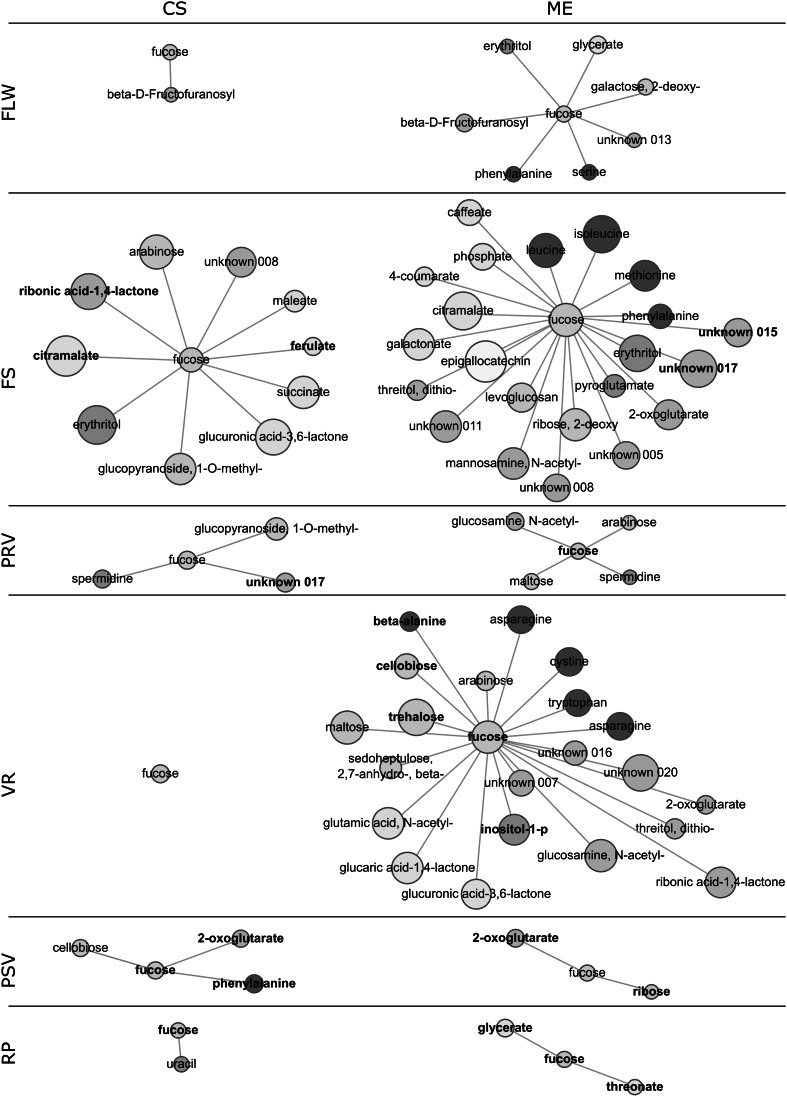
Neiborhood of fucose according to the reconstructed networks shown in Supplementary Fig. 6 for Cabernet Sauvignon (CS) and Merlot (ME) and for each developmental stage (FLW-RP). In each network, nodes represent metabolites and edges depict significant positive (*blue*) or negative (*red*) correlations (|*r*| > 0.75, *p* < 0.001). Metabolites with significant changes (*p* < 0.05) respect to the first stage (FLW) are formatted in *bold font*. Node size is proportional to its degree and its color represent metabolite classes (according to Table 1) (Color figure online)

One unexpected result of this analysis is that the most abundant sugars during berry development, such as glucose, fructose and sucrose, do neither exhibit high degrees nor high betweenness coefficients in nearly all networks for both cultivars (see Supplementary Table 7B), with the notable exceptions of glucose in CS-veraison and fructose in ME-veraison. Fucose, on the other hand, seems to be a very important component of every network in ME, due to its high degree and betweenness (see Supplementary Table 7 among top 20 metabolites), which can also be observed in Fig. 5, where its neighborhood is illustrated. This prominent position of fucose in case of CS was only conserved for the fruit-setting and pre-veraison stages (Fig. 5, Supplementary Table 7A). Cellobiose, glucose-6-p and trehalose showed high betweenness and degree coefficients during the early stages of development in both cultivars with cellobiose displaying the highest betweenness for the flower stage. Trehalose and glucose-6-p sugar presented again high betweenness during later stages, but at different developmental stages for the two cultivars (post-veraison in CS and veraison ME, in Supplementary Table 7).

Amino acids show high betweenness and degree coefficients at the early stages for both cultivars, namely flowering and fruit settings. For most amino acids these coefficients decreased during later stages, with the exceptions of a few amino acids that showed very high betweenness such as alanine in ME pre-veraison (see Supplementary Fig. 8A), phenylalanine in CS-post-veraison (see Supplementary Fig. 8B), and methionine in CS-veraison (see Supplementary Fig. 8C). In ripening, a cluster of amino acids was obtained in CS, having isoleucine as central node (see Supplementary Fig. 9A), which was not observed in ME, where amino acids were dispersed (see Supplementary Fig. 9B).

## Discussion

### Primary metabolite changes allow for the discrimination between different developmental stages of grapes of a given cultivar

The application of an untargeted metabolomics approach based on GC–MS to study grape berry development has allowed the identification and quantification of 115 metabolites during different stages from flowering until ripening of two grapevine cultivars. This large-scale comparative study provides a more comprehensive view on the major and important pathways related to primary metabolism which may be involved in fruit development, thus offering a better understanding of berry development and ripening biochemistry. The results obtained from the analysis of two cultivars, used worldwide for wine production, contribute with novel information and complements the limited data available by using untargeted metabolomic approaches (Toffali et al. 2011, Torniellei et al. 2012; Ali et al. 2011; Dai et al. 2013; Agudelo-Romero et al. 2013a; Son et al. 2014; Teixeira et al. 2014) for dilucidating metabolic changes involved in growth, development and ripening of grapevine berries.

The metabolism analysis of both varieties (CS and ME) revealed that grape cultivars undergo several changes in primary metabolite concentration during berry developmental progression. The content and presence of primary metabolites alone enable to discriminate between the grape developmental stages of a given cultivar, as demonstrated by PCA in which the earliest stages can be clearly distinguished from the latest ones (Fig. 1), or between both cultivars at a determined stage as shown by PLS (see Supplementary Fig. 3). This pattern was observed in both grape cultivars. The putative biomarkers, discriminating the developmental stages of both cultivars, were observed to either increase or decrease in abundance at specific developmental phases. Thus, asparagine, phenylalanine, rhamnose and unknown metabolite 15 act as the best discriminating factors for early stages whereas allo-inositol and the unknown metabolite 9 might represent markers to separate late stages such as ripening. Well-known substances accumulated at particular developmental steps and described as important players in biochemical and physiological processes of grapevine berries like glucose, malate and tartrate (Ruffner and Hawker 1977; Davies and Robinson 1996; Robinson and Davies 2000; Coombe and McCarthy 2000; Conde et al. 2007; Martínez-Esteso et al. 2013) allow for the discrimination between stages within a cultivar (the levels of glucose and fructose increase, and are one of the main contributors in the PCA Fig. 1b), but they do not enable us to differentiate between cultivars at a given stage (e.g., FLW-CA vs FLW-ME, Fig. 1a).

### The main changes in primary metabolites occur in fruit setting and post-veraison in both cultivars

Another similarity between the cultivars was the number of significant metabolite changes between developmental stages, which increased during the growth period in comparison to the first developmental stage and exhibited a similar pattern when the comparison was performed between continuous stages (Fig. 3). The initial chemical composition detected in both cultivars at flowering period undergoes variations during the different stages until ripening. However, the larger changes in metabolite concentration occur in fruit setting and post-veraison in both cutlivars (Fig. 3a and b). Fruit setting is characterized by the beginning of sigmoidal growth phase and cell division. Similarly, post-veraison is characterized by the resume of growth, softening of berries and accumulation of sugars (Coombe and McCarthy 2000; Dai et al. 2013), which involve several biochemical and physiological modifications associated to changes in the levels (increasing or decreasing) of particular metabolites. Our results demonstrate that significant metabolite changes occur at specific stages of the development of grapevine berries and such metabolic changes are expressed mainly by a down regulation of different substances related to primary metabolism.

Many of the primary metabolites that changed their concentration have been already extensively described in the literature. For instance, sugars showed a significant increase beginning with veraison in both cultivars. Malic acid peaked its concentration around veraison and started to decrease in post-veraison and ripening (Conde et al. 2007). Tartaric acid exhibits a decrease in concentration around veraison in both cultivars (Pilati et al. 2007). Amino acids (serine, valine, leucine, isoleucine) displayed a peak in flowers and strong decrease in post-veraison and ripening, while asparagine, proline and β-alanine showed a increase in post-veraison and in ripening. This seems to be different from other reported studies in another Vitis species, in *Vitis rotundifolia*, where amino acids contents peaked in veraison, although proline and β-alanine showed a consistent behavior (Lamikanra and Kassa 1999). Our results demonstrated that the metabolites content detected at early stages (flowering) in both cultivars is altered during the developmental and ripening process and that the main changes are related to a reduction (down regulation) of their amounts. This suggests that a catabolic activity is acting upon these metabolites leading to a reduction on their levels which may be required to allow the action of other metabolic mechanism for the production of other classes of molecules needed for development and ripening of the berries. Thus, the content of different classes of sugars, aminoacids and polyamines are reduced as the course of maturation progresses (Fig. 4). Interestingly, levels of polyamines like spermidine and spermine are higher at earlier stages (flowering) and decreased during grape ripening in both CS and ME. Similar results have been recently reported for the development of Trincadeira berries where the reduction of polyamines levels is accompanied by an up-regulation of genes involved in the catabolism of polyamines suggesting a role of polyamine catabolism in grape ripening (Agudelo-Romero et al. 2013b, 2014).

### Network analysis exhibited different numbers of edges, density, and other network topology parameters depending on the developmental stage and on the cultivar

We described a metabolite CNs analysis for cultivars CS and ME in every developmental stage in order to gain a broader insight into how the measured metabolites are related to each other in both cultivars during the berry growth process. CN enables the integration of information of diverse backgrounds (e.g., metabolites, physiological traits or genes), considering key features allowing for the analysis of coordinated changes of metabolites based on correlation coeficients. CN has become an increasingly popular tool to represent the relationships of metabolites (Toubiana et al. 2013; Hochberg et al. 2013). The main observation was that the degree distribution of all networks followed a power-law, which is a intrinsic property of “scale-free” networks (Albert and Barasi 2000; Arita 2005; Watts and Strogatz 1998) in which few nodes (so-called *hubs*) are responsible for most of the network connectivity. This allows us to identify important primary metabolites of a network by looking at their degree. In addition to that, we also identified metabolites with high betweenness centrality. Such nodes may be regarded as important for transport and communication between disjoint sections of a network (Martin-Gonzalez et al. 2010). Another result was that the metabolite networks exhibited different numbers of edges, density, and other network topology parameters dependending on the developmental stage and on the cultivar, which may suggest different regulatory mechanism (Fig. 5a). For instance, in CS a marked increase in the coordinated metabolic activities is observed mainly in one specific stage (fruit setting) whereas in ME a very dense network can be observed in fruit setting and in veraison. Furthermore, the CN analysis highlighted the structural role of central metabolites. For instance, most amino acids displayed high degree and betweenness in the early developmental stages, specifically during flowering and fruit-setting. Glucose and fructose exhibited relatively high betweenness only during veraison in CS and ME. This might be related with the fact that the latter takes more time to ripe, and the accumulation of sugar might have started later. The network approach does not rely on the identification of metabolite concentration changes, but rather on the network properties of the metabolite–metabolite interaction, which might complement the information obtained with the previous (classical) approach. The increased network density and connectedness observed at certain stages, specifically at fruit setting in both cultivars, shows that fucose is playing an important connecting role in both cultivars with a higher association grade in ME (flowering, fruit setting and veraison) than in CS (mainly in fruit setting) (Fig. 5b).

It is of interest to note that significant changes of fucose were not observed in any of the developmental stages, in particular during fruit-setting, were its concentration was around average in both cultivars (Supplementary Fig. 5B), but in contrast it showed a high node degree in both cultivars, which might mean that fucose is an essential metabolite in that stage (Jeong et al. 2001). This finding would have not been possible just by looking at concentration changes. Fucose is a hexose deoxy sugar with the chemical formula C_6_H_12_O_5_ that is present in a wide variety of organisms and has been shown its role on different biological events (Becker and Lowe 2003; Wijesinghe and Jeon 2012). Different studies have reported the influence of fucose obtained from algae on plant defense mechanism enhancing protection against pathogens (virus, fungi) and acting as an elicitor by activating salycilic acid, jasmonic acid and ethylene signaling pathways at systemic level (Chong et al. 2002; Klarzynski et al. 2003; Sels et al. 2008; Vera et al. 2011). If this metabolite is playing a role in the regulation of primary metabolites changes during grape berry development remains to be demonstrated.

Recently, studies using a systems strategies for combining plant transcriptome and GC–MS metabolomic data to develop associations in tomato (Mounet et al. 2009; Enfissi et al. 2010; Osorio et al. 2011) or grape (Carrari et al. 2006; Deluc et al. 2007; Zamboni et al. 2010; Osorio et al. 2011; Dai et al. 2013) have provided novel insights into the crucial influence of changes in primary metabolites and on fruit ripening and quality, allowing the identification of similar and distinct regulation at the gene and metabolite levels between nonclimateric and climateric fruits (Osorio et al. 2012; Biais et al. 2014). The combination of this information with metabolite levels provides a better understanding about relationships between metabolism, fruit development, and maturation. Correlation-based network analysis highlighted a dense degree of connectivity, building stage-specific metabolic modules, mainly during early to mid grapevine berry ripening similar to other nonclimateric (strawberry; Fait et al. 2008) or climateric (tomato pericarp: Ursem et al. 2008) fruits. The elements (metabolites) allowing such degree of connectivity are probably different across the cultivars, but this type of evidence suggests some common mechanism among nonclimateric and climacteric fruit at the basis of metabolic regulation involving a high connectivity of primary metabolites mainly in early developmental stages. Furthermore, application of new strategies for analysis of publicly available metabolomics data from nonclimateric and climateric fruits as STATIS (an extension to PCA combined with pathway overenrichment analysis) has allowed the identification of metabolic processes whose behavior is similarly affected during fruit development and maturation across species (Klie et al. 2014).

One of the disadvantages of using total correlation, as performed in our study, is that it is not possible to distinguish direct and indirect interactions between two metabolites, in other words, a significant correlation between them may originate due to the interaction of a third metabolite and not due an actual direct relationship. A method to circumvent this problem is partial correlation, defined as the correlation of two variables conditioned to a third variable (De la Fuente et al. 2004), which may help in excluding such indirect interactions. Although partial correlation does not reveal causal relationships, this approach could be a potential improvement to our study towards the uncovering of the true metabolite interactions.

## Concluding remarks

*Vitis vinifera* cultivars undergo a highly coordinated metabolic shift of metabolites associated to primary metabolism during the stages involved in growth, development and ripening of berries. The changes are characteristics for each stage, the most pronounced ones occuring at fruit setting and pre-veraison. Most of the changes are associated to a reduction of the levels of the metabolites present in the earlier corresponding stage revealing a required catabolic activity of primary metabolites to allow grape berry ripening and synthesis of other types of molecules. Network analysis demonstrated that the network connectivity of primary metabolites depends on stage and cultivar, suggesting differences in metabolism regulation in CS and ME as the maturity processes progress. Furthermore, network analysis represents an appropriate method to display the association between primary metabolites during berry developmental processes among different grapevine cultivars and for identifying potential biologically relevant metabolites.

## Electronic supplementary material

Supplementary material 1 (PDF 830 kb)

Supplementary material 2 (XLSX 1201 kb)

Supplementary material 3 (XLSX 13 kb)

Supplementary material 4 (XLSX 18 kb)

Supplementary material 5 (XLSX 12 kb)

Supplementary material 6 (XLSX 19 kb)

Supplementary material 7 (XLSX 11 kb)

Supplementary material 8 (XLSX 17 kb)

Supplementary material 9 (XLSX 34 kb)
