# Supplementary material for: GC–MS metabolic profiling of Cabernet Sauvignon and Merlot cultivars during grapevine berry development and network analysis reveals a stage- and cultivar-dependent connectivity of primary metabolites
Source: Metabolomics. 2016 Jan 23;12:39. doi: 10.1007/s11306-015-0927-z (PMC4723623; doi:10.1007/s11306-015-0927-z)

SUPPLEMENTARY INFORMATION

GC-MS metabolic profiling of Cabernet Sauvignon and Merlot cultivars during grapevine berry development and network analysis reveals a stage- and cultivar-dependent connectivity of primary metabolites

Alvaro Cuadros-Inostroza[1,2], Simón Ruíz-Lara[3], Enrique González[3], Aenne Eckardt[1], Lothar Willmitzer[3], and Hugo Peña-Cortés[1,4*]

[1]Max-Planck Institute for Plant Molecular Physiology, Am Mühlenberg 1, 14476. Potsdam-Golm Golm, Germany.
[2]MetasysX, Am Mühlenberg 11, 14476. Potsdam-Golm Golm, Germany (Current Address).
[3]Instituto de Ciencias Biológicas, Universidad de Talca, 2 Norte 685, Talca, Chile.
[4]Facultad de Medicina, Hontaneda 2653, Universidad de Valparaíso de Chile, Valparaíso 2340000, Chile.

Corresponding author*:
Hugo Peña-Cortés
Facultad de Medicina, Hontaneda 2653, Universidad de Valparaíso de Chile, Valparaíso 2340000, Chile.
Email: hugo.pena@uv.cl
Phone/FAX: +56 32 250732

Supplementary Figure 1. Grapevine developmental growth curve. Fresh weight of grape samples from flowering stages until mature (ripening) berries from the two cultivars selected were collected during two different seasons. Sampling was carried out consistently at approximately 10:00 a.m. to discard possible oscillations in metabolite levels. Corresponding to their developmental stage, collected clusters were broken down into berries before analysis. Phenological stages analyzed and their corresponding numbers according to the 'Modified Eichorn-Lorenz classification system' (Coombe 1995) were: Flowering (EL-23); Fruit setting,(EL-29), Pre-veraison,(EL-33); Veraison, (EL-35); Post-veraison, (EL.36) and Ripening (EL- 38). Five biological replicates of around 10-15 g of material for each stages and in each season were collected. Adapted from (Kennedy 2002).

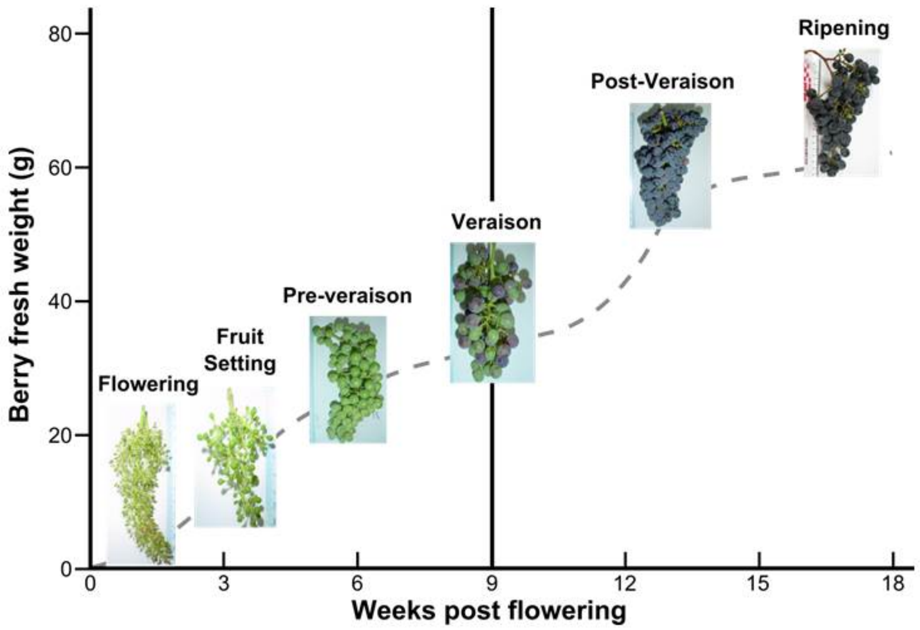

Supplementary Figure 2. Flowchart of the network analysis. We performed metabolite-metabolite correlation within each stage in order to obtain a correlation matrix for all developmental stages. Then, a permutation test was applied in order to obtain a r-threshold at a significance level of 0.001. Using this threshold, a network was derived from the corresponding adjacency matrix. Network operations were perfomed using the package igraph and network visualization with cytoscape as indicated. For the rest of computation, custom R-scripts were used.

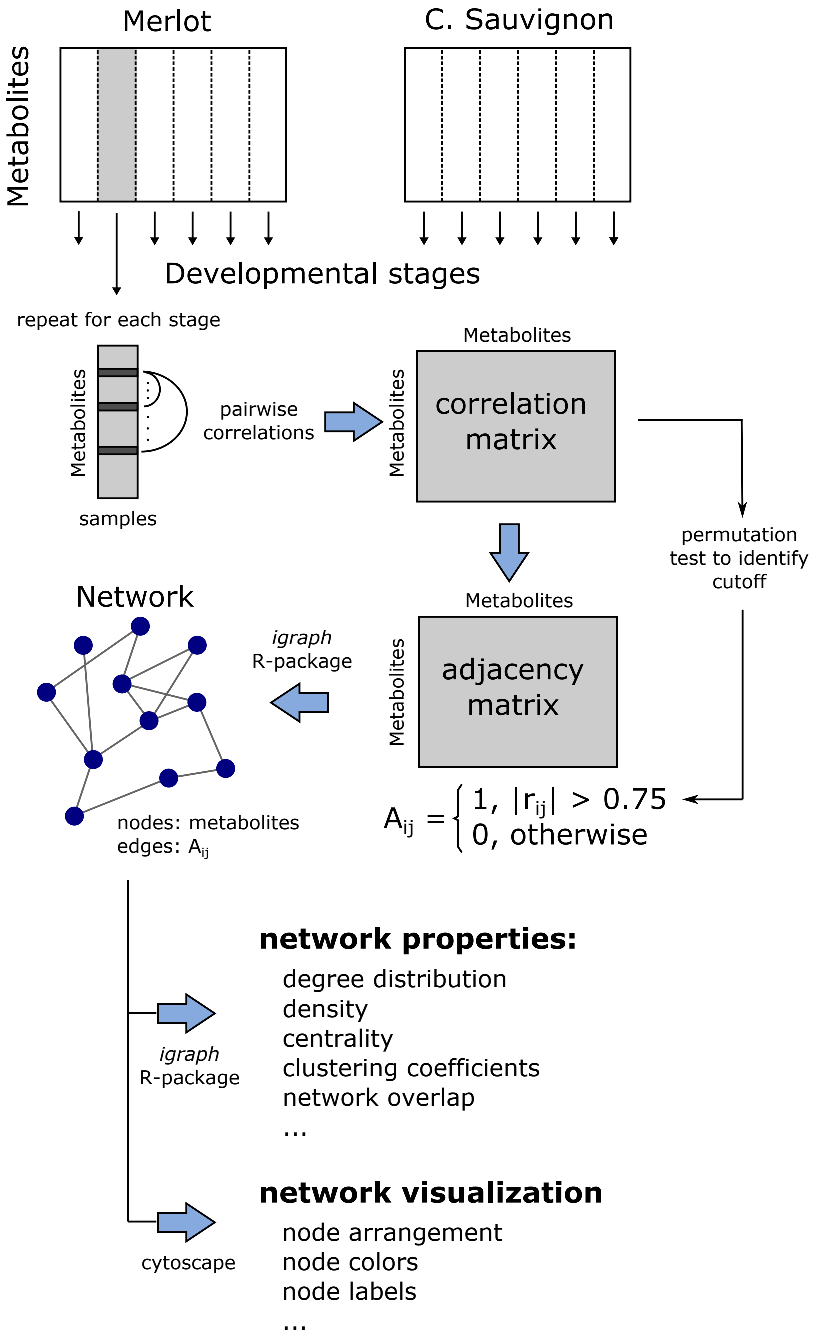

Supplementary Figure 3. (A) Partial Least Squares (PLS) representation of each Cabernet Sauvignon (CS) and Merlot (ME) samples for a particular developmental stages: flowers, fruit setting, pre-veraison, veraison, post-veraison and ripening. The explained variances of PLS components are shown in the upper-right corner. (B) Error rate of classification error for each developmental stage. The error was estimated by leave one out cross-validation.

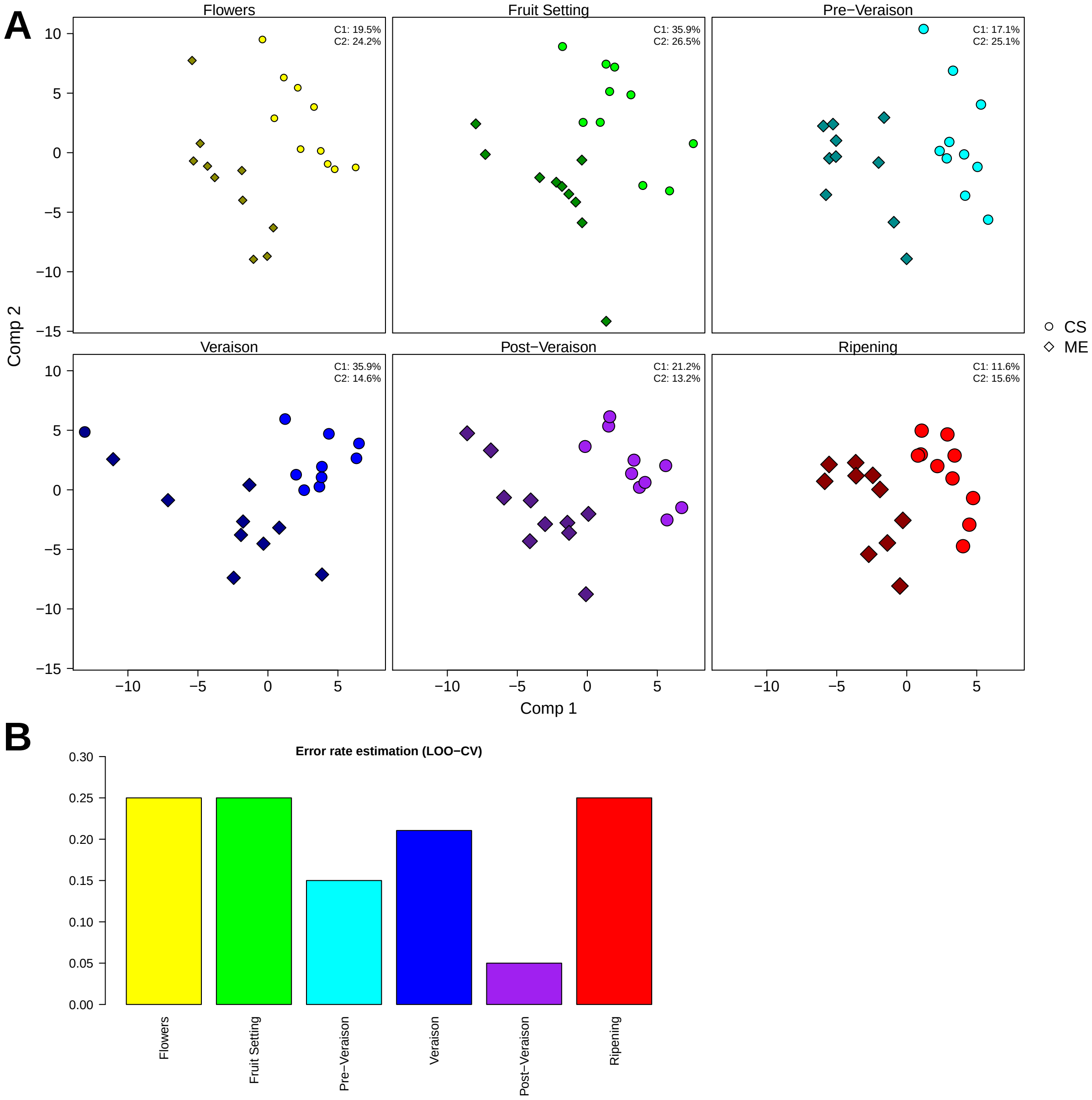

Supplementary Figure 4. Hierarchical Clustering Analysis (HCA) of primary metabolites of grapes samples. In the heat map, rows represent metabolites as indicated and columns are samples. Log_2-transformed metabolite intensities are color coded as shown in the gradient scale above, whilst missing values are colored in white. The samples are divided in two groups according to cultivar (left panel: Cabrenet Sauvignon, CS; right panel: Merlot, ME), and ordered from left (earliest growth stage) to right (latest development stage).

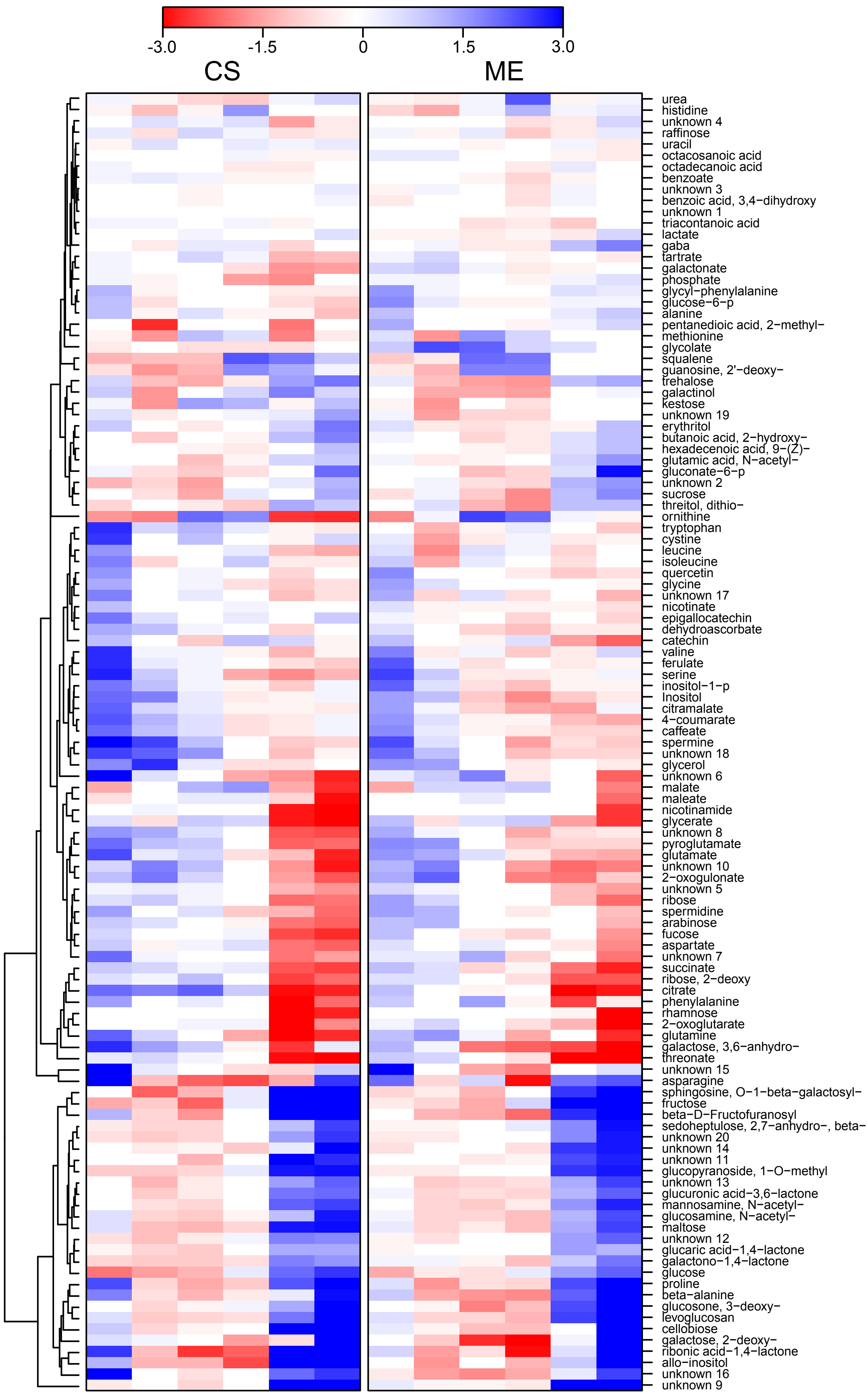
-3.0
-1.5
0
1.5
3.0
CS
ME
urea
histidine
unknown 4
raffinose
uracil
octacosanoic acid
octadecanoic acid
benzoate
unknown 3
benzoic acid, 3,4−dihydroxy
unknown 1
triacontanoic acid
lactate
gaba
tartrate
galactonate
phosphate
glycyl−phenylalanine
glucose−6−p
alanine
pentanedioic acid, 2−methyl−
methionine
glycolate
squalene
guanosine, 2'−deoxy−
trehalose
galactinol
kestose
unknown 19
erythritol
butanoic acid, 2−hydroxy−
hexadecenoic acid, 9−(Z)−
glutamic acid, N−acetyl−
gluconate−6−p
unknown 2
sucrose
threitol, dithio−
ornithine
tryptophan
cystine
leucine
isoleucine
quercetin
glycine
unknown 17
nicotinate
epigallocatechin
dehydroascorbate
catechin
valine
ferulate
serine
inositol−1−p
Inositol
citramalate
4−coumarate
caffeate
spermine
unknown 18
glycerol
unknown 6
malate
maleate
nicotinamide
glycerate
unknown 8
pyroglutamate
glutamate
unknown 10
2−oxogulonate
unknown 5
ribose
spermidine
arabinose
fucose
aspartate
unknown 7
succinate
ribose, 2−deoxy
citrate
phenylalanine
rhamnose
2−oxoglutarate
glutamine
galactose, 3,6−anhydro−
threonate
unknown 15
asparagine
sphingosine, O−1−beta−galactosyl−
fructose
beta−D−Fructofuranosyl
sedoheptulose, 2,7−anhydro−, beta−
unknown 20
unknown 14
unknown 11
glucopyranoside, 1−O−methyl
unknown 13
glucuronic acid−3,6−lactone
mannosamine, N−acetyl−
glucosamine, N−acetyl−
maltose
unknown 12
glucaric acid−1,4−lactone
galactono−1,4−lactone
glucose
proline
beta−alanine
glucosone, 3−deoxy−
levoglucosan
cellobiose
galactose, 2−deoxy−
ribonic acid−1,4−lactone
allo−inositol
unknown 16
unknown 9

Supplementary Figure 5. Main HCA clusters of primary metabolites of grapes samples (A-C). In each heat map, rows represent metabolites as indicated and columns are samples. Log_2-transformed median centered metabolite intensities are color coded as shown in the gradient scale above, while missing values are colored in white. The samples are divided in two groups according to cultivar, and ordered from left (earliest growth stage) to right (latest development stage). Flowerings (FLW), fruit setting (FS), pre-veraison (PRV), veraison (VR), post-veraison (PSV) and ripening (RP). See Supplementary Fig.4 for the full heatmap.

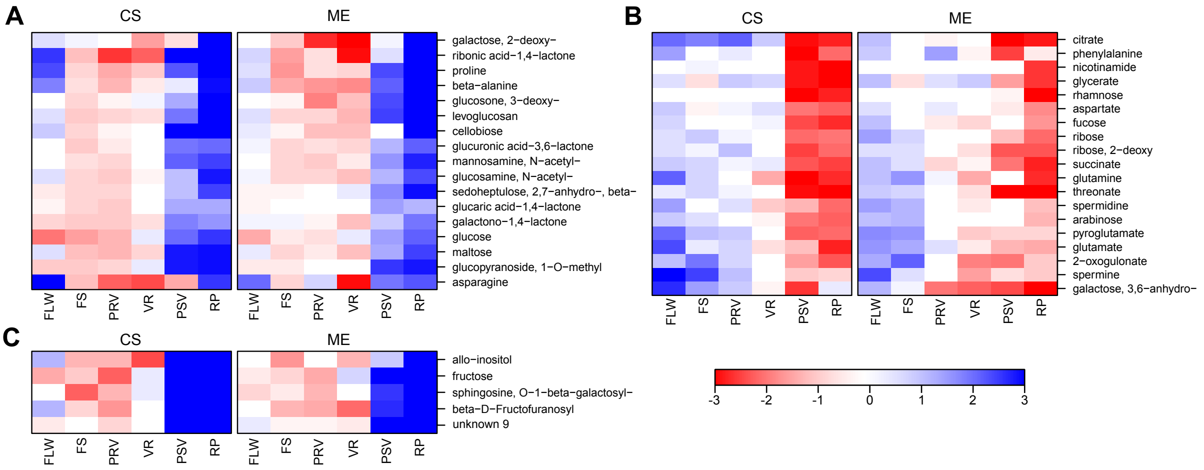

Supplementary Figure 6. Common and unique significant changing metabolites between cultivars Cabernet Sauvignon and Merlot respect to (A) the initial stage (flowering, FLW) and (B) the previous stage (see also Fig. 3). The color of the numbers in each venn diagram indicates whether metabolites increases (blue) or decreases (red). The significance of the metabolite overlap is indicated with stars (*< 0.1, **< 0.01, ***< 0.001).

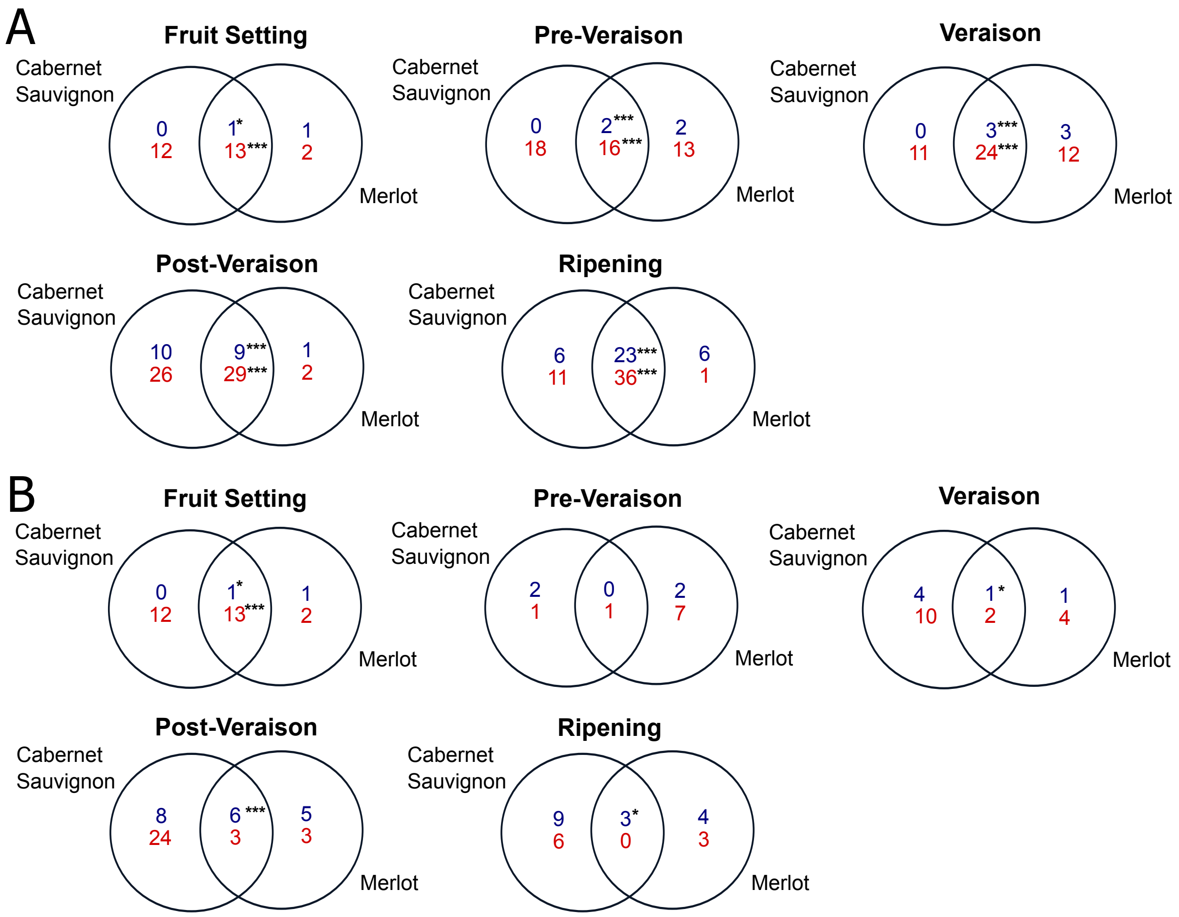

Supplementary Figure 7. Overview of primary metabolite networks for Cabernet Sauvignon (CS) and Merlot (ME). Networks for each grape developmental stage flowerings (FLW), fruit setting (FS), pre-veraison (PRV), veraison (VR), post-veraison (PSV) and ripening (RP) are shown. In each network, nodes represent metabolites and edges significante positive (blue) or negative correlations (red) ($|r| < 0.75$, $p < 0.001$). Node size is proportional to its degree and its color represents metabolite classes (according to Table 1). Metabolites with significant changes ($p < 0.05$) respect to the first stage (FLW) are depicted with bold font. Isolated vertices are not shown.

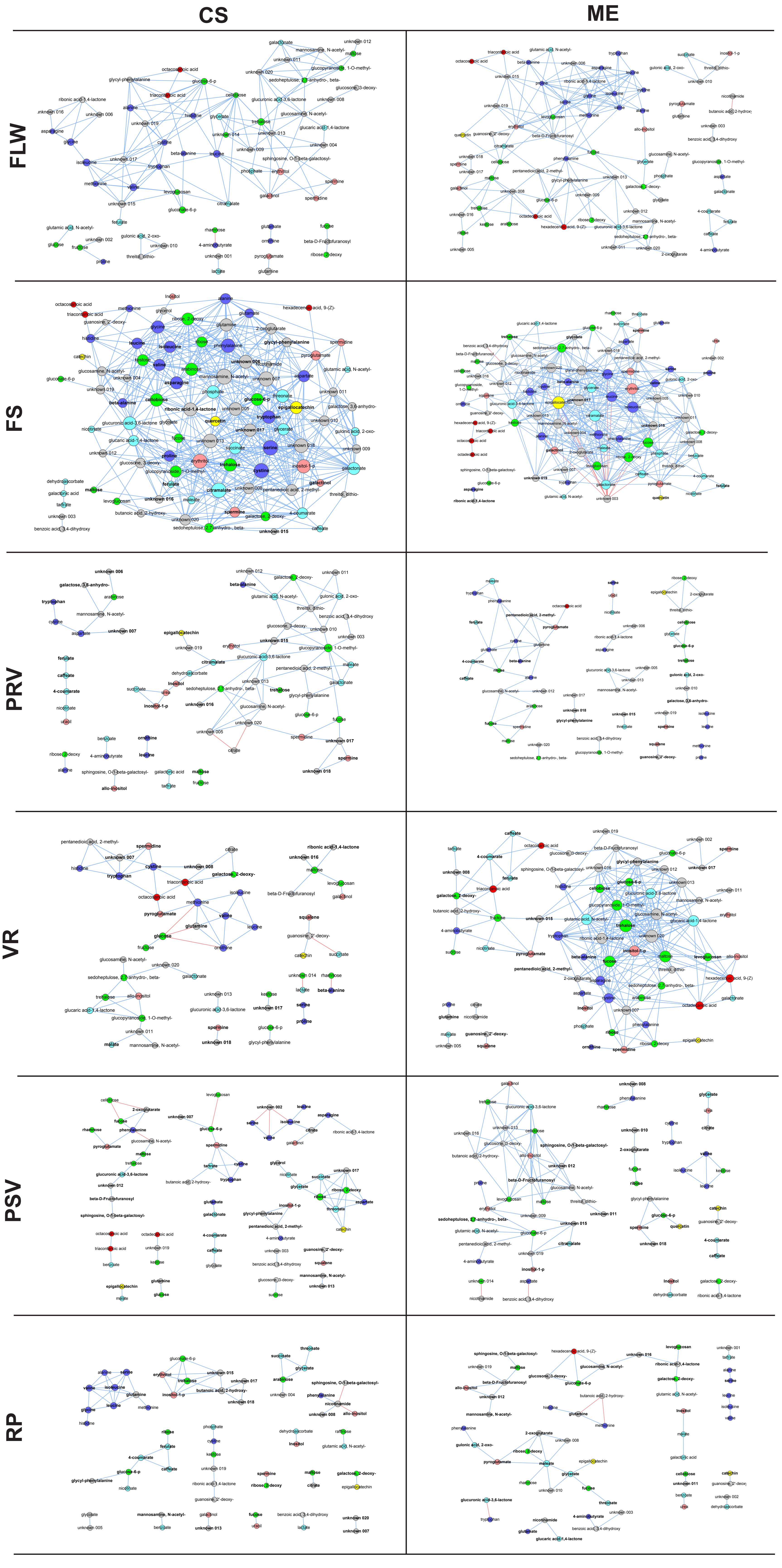
CS
ME
FLW
FS
PRV
VR
PSV
RP

Supplementary Figure 8. Metabolite correlation network neighborhood of amino acids that exhibited high betweenness coefficients. (A) alanine in Merlot pre-veraison, (B) phenylalanine in Cabernet Sauvignon post-veraison, and (C) methionine in Cabernet Sauvignon veraison. Size of the node is proportional to its degree. Vertices are color coded according metabolite classes (Table 1) as illustrated. Positive and negative correlations are indicated by blue and red lines, respectively. Metabolites that changed significantly with respect to the stage flowers are in bold font.

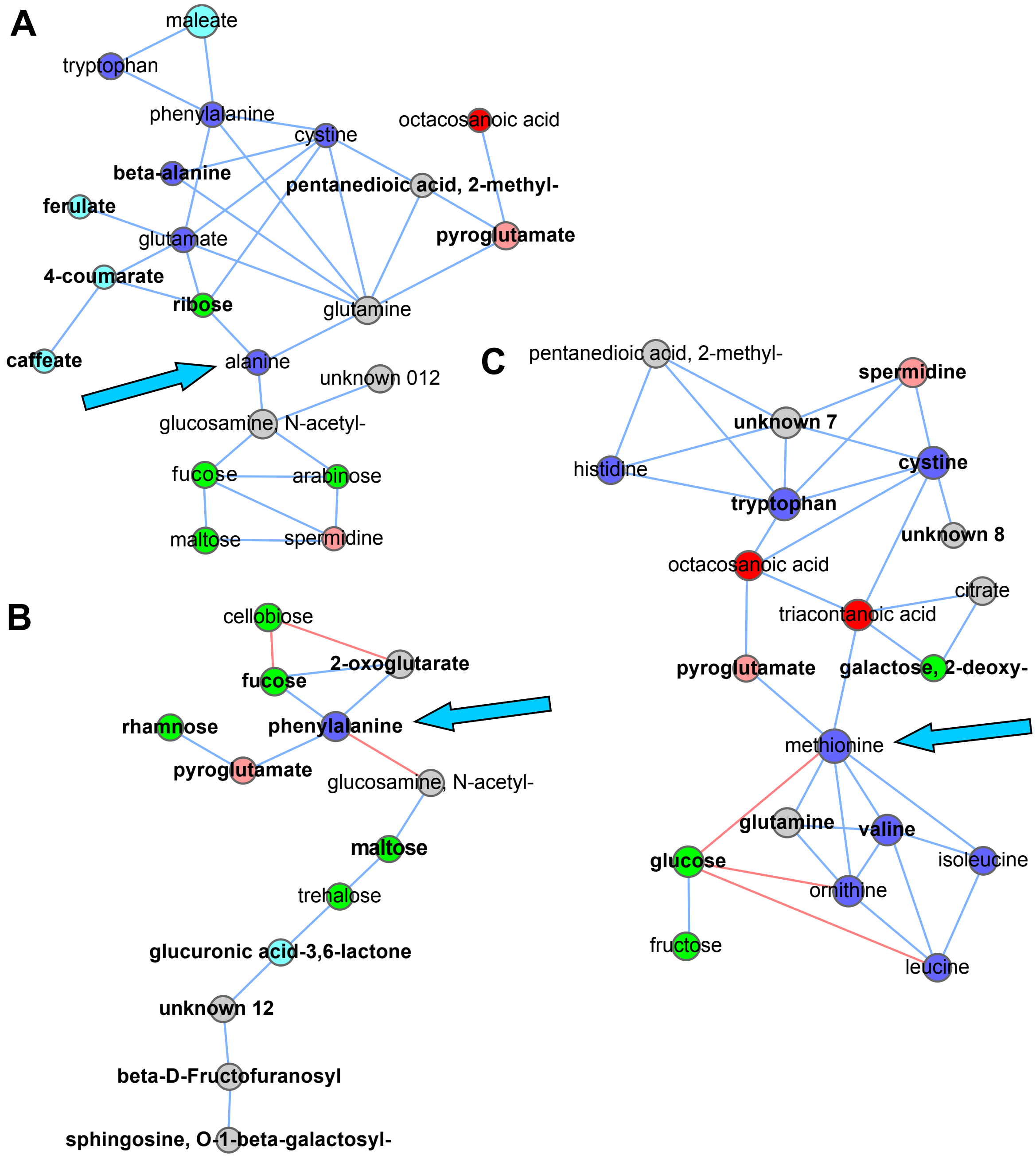

Supplementary Figure 9. Metabolite correlation network of amino acids during ripening in Cabernet Sauvignon (A) and Merlot (B). Blue nodes are amino acids and gray nodes are unclassified metabolites. Positive and negative correlations are indicated by blue and red lines, respectively. Metabolites that changed significantly with respect to the stage flowers are in bold font.

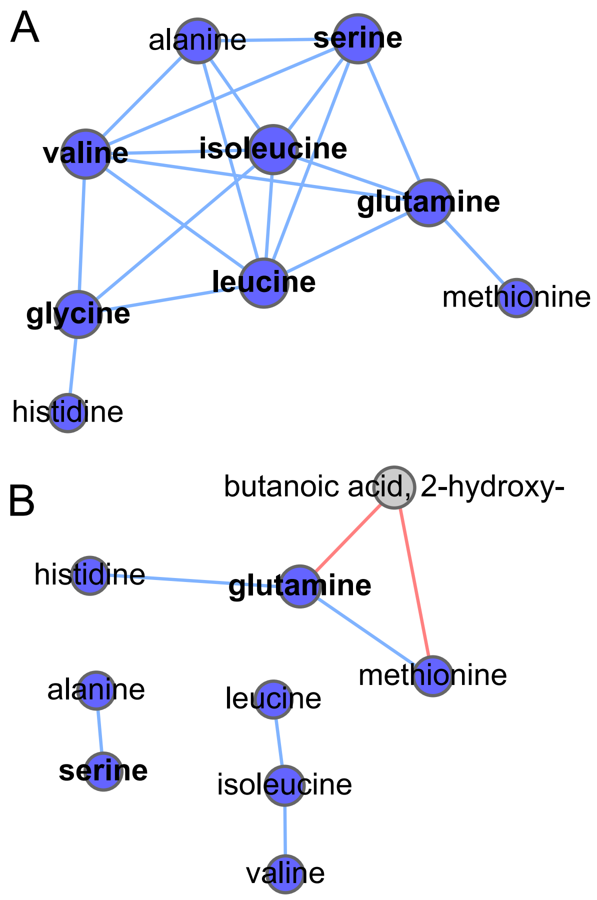

Supplement: Supplementary file 1 — Supplementary material 1 (PDF 830 kb) [file 11306_2015_927_MOESM1_ESM.pdf]
